# 4401 Incidence, management, and outcomes of immune-related adverse events (irAEs): an analysis of a multidisciplinary toxicity team for cancer immunotherapy related irAEs

**DOI:** 10.1017/cts.2020.239

**Published:** 2020-07-29

**Authors:** Aanika Balaji, Jiajia Zhang, Jarushka Naidoo

**Affiliations:** 1 Johns Hopkins University School of Medicine

## Abstract

OBJECTIVES/GOALS: This study aims to assess the outcomes of a new virtual multidisciplinary immune-related toxicity (IR-tox) team implemented at Johns Hopkins Hospital. In particular, to understand if the IR-tox team’s input reduced the number of inpatient hospitalizations for irAEs for referred patients. METHODS/STUDY POPULATION: Since August 2017, nearly 250 patient referrals to the IR-tox team have been created and stored in an electronic database. Through retrospective chart review, hospitalization and irAE management data will be collected for these patients to assess whether rates for suspected irAEs have decreased. These rates will be compared against historical controls. We will assess the features of hospitalized patients, their immunotherapy regimens, and management to identify high-risk groups who may require early intervention. Additionally, we aim to understand what patient features are associated with IR-Tox team referral and subsequent hospitalization. RESULTS/ANTICIPATED RESULTS: The IR-tox team provided a new multidisciplinary channel to help physicians diagnose and manage complex irAEs. The goal of the team was the reduce the number of irAE-related hospitalizations as, historically, 85% of high-grade irAEs have required hospitalization. A clinically meaningful reduction is defined as lowering the hospitalization rate to 75%. Planned analyses includes calculating the hospitalization rate, using descriptive statistics to summarize patient features, multivariate analyses to understand features associated with both IR-Tox team referral and hospitalization, and computing the relative risk reduction to assess the efficacy of subspecialist referral implementation. DISCUSSION/SIGNIFICANCE OF IMPACT: IrAEs are challenging to diagnose and treat. They contribute to a notable proportion of hospitalizations in those treated with immunotherapy. With expanding use of immunotherapy, widespread implementations of IR-Tox teams may help reduce hospitalizations and costs associated with care for irAEs.

